# Error monitoring in musicians

**DOI:** 10.3389/fnhum.2013.00401

**Published:** 2013-07-26

**Authors:** Clemens Maidhof

**Affiliations:** ^1^ Cognitive Brain Research Unit, Cognitive Science, Institute of Behavioural Sciences, University of HelsinkiHelsinki, Finland; ^2^ Finnish Centre of Excellence in Interdisciplinary Music Research, University of JyväskyläJyväskylä, Finland

**Keywords:** EEG, performance monitoring, music performance, motor control, errors

## Abstract

To err is human, and hence even professional musicians make errors occasionally during their performances. This paper summarizes recent work investigating error monitoring in musicians, i.e., the processes and their neural correlates associated with the monitoring of ongoing actions and the detection of deviations from intended sounds. Electroencephalography (EEG) studies reported an early component of the event-related potential (ERP) occurring before the onsets of pitch errors. This component, which can be altered in musicians with focal dystonia, likely reflects processes of error detection and/or error compensation, i.e., attempts to cancel the undesired sensory consequence (a wrong tone) a musician is about to perceive. Thus, auditory feedback seems not to be a prerequisite for error detection, consistent with previous behavioral results. In contrast, when auditory feedback is externally manipulated and thus unexpected, motor performance can be severely distorted, although not all feedback alterations result in performance impairments. Recent studies investigating the neural correlates of feedback processing showed that unexpected feedback elicits an ERP component after note onsets, which shows larger amplitudes during music performance than during mere perception of the same musical sequences. Hence, these results stress the role of motor actions for the processing of auditory information. Furthermore, recent methodological advances like the combination of 3D motion capture techniques with EEG will be discussed. Such combinations of different measures can potentially help to disentangle the roles of different feedback types such as proprioceptive and auditory feedback, and in general to derive at a better understanding of the complex interactions between the motor and auditory domain during error monitoring. Finally, outstanding questions and future directions in this context will be discussed.

## Introduction

Research into errors occurring during skilled human behavior has a long tradition, and has revealed important insights into planning and execution of complex tasks, such as speech, typing, and music performance (for the music domain, see e.g., Palmer and van de Sande, 1993, 1995; Palmer, 1997, 2005; Palmer and Pfordresher, 2003; Pfordresher, 2006). From the perspective of the experienced performer, errors (defined as the unintended result of an action) are usually something that has to be avoided. Nevertheless, even highly trained musicians commit errors occasionally. In contrast, errors provide an important source of information during music learning. Only if we are aware of our errors, e.g., when we realize that a keystroke sounded wrong (whether in terms of pitch, timing, or intensity), we can learn and try to prevent them in the future. Thus, the constant monitoring of our actions, detecting errors, and appropriately responding after we have realized that our actions did not result in what was intended is an important feature of human behavior.

Early investigations of errors during skilled behavior such as typing (Rabbitt, 1978) showed that participants can detect their own errors immediately, possibly even before the result of an erroneous action can be perceived. In addition, participants showed slower responses on trials following errors (“post-error slowing”), suggesting that they adjusted their behavioral strategies after error commissions. Consequently, it was assumed that the functioning of an action-monitoring system is responsible for the detection of errors and initiation of performance adjustments when appropriate.

The neurophysiological correlates of erroneous responses, compared to correct responses, were investigated in the 1990s. A seminal finding was a sharp, negative-going deflection in the ERP occurring around 50–100 ms after participants responded incorrectly during choice-reaction tasks (for reviews, see e.g., Falkenstein et al., 2000; van Veen and Carter, 2006; Taylor et al., 2007). This component, termed “Error Negativity” (Ne) (Falkenstein et al., 1990) or “error-related negativity” (ERN) (Gehring et al., 1993) seemed to be elicited independently of the modality in which the stimulus is presented in (Falkenstein et al., 2000), and independently of the effector (hand or foot) with which the erroneous response was made (Holroyd et al., 1998). Converging evidence from source localization of EEG data (e.g., van Veen and Carter, 2002; Herrmann et al., 2004), primate studies (e.g., Gemba et al., 1986), and functional magnetic resonance imaging (fMRI) (e.g., Ullsperger and von Cramon, 2001; Debener et al., 2005) indicate that the ERN is generated in the anterior cingulate cortex (ACC) (for a review, see Ridderinkhof et al., 2004). Several theories try to account for the ERN findings: one of the first hypotheses was that the ERN is induced when the comparison between the neural representation of the correct response with the representation of the actual response shows a mismatch (Falkenstein et al., 2000). The reinforcement-learning theory posits that the ERN is elicited whenever an outcome (based on a response or on the feedback given to participants) is worse than expected (Holroyd and Coles, 2002), and the conflict monitoring theory assumes that the ERN is elicited when the ACC detects conflict due to the simultaneous activation of two competing response representations (Carter et al., 1998; Botvinick et al., 2001; van Veen et al., 2001).

However, the neural correlates of errors during the performance of time-based sequential behaviors like music remained largely elusive. In the following, recent approaches in this domain (focusing on piano performance) and their findings will be summarized.

## Errors during music performance

Many errors committed during the performance of a complex piece of music go unnoticed by the listener, presumably because of the context they appear in, their loudness, and whether the listener is familiar with the piece of music or not (Repp, 1996). Although the relevance of an error that is not perceived by a listener is, for the purpose of musical communication, arguable, errors can be identified on a more objective level. This can be realized because a great deal of music in the western traditional music culture is passed on in a written form (i.e., musical scores), and the identification of errors can be achieved by comparing the notation with the actually performed notes. When the performance is compared to a score, at least three different types of errors can be differentiated: substitutions occur when a note is performed with a wrong pitch, omissions occur when a note is not performed at all, and intrusions (or additions) occur when a note is played that is not in the score (although further distinctions within these types can be made, and error coding can be ambiguous; Palmer and van de Sande, 1993; Repp, 1996). In addition to these errors, musicians can make mistakes during fingering, e.g., when a note is produced with a different finger than originally intended (the problem of fingering is especially pronounced with keyboard instruments, because a clear finger-key mapping is missing). These fingering errors will usually not directly result in a wrong or missing note; however, they might cause problems in motor planning and execution of the following events, possibly resulting in a substitution, deletion, or addition at later positions in a performance.

Recently, a couple of studies using EEG started to investigate the neural correlates of such substitution errors during piano performances (Maidhof et al., 2009; Ruiz et al., 2009, 2011; Strübing et al., 2012). The study of neural correlates of errors can provide insights into the mechanisms of human action monitoring during a complex, multimodal task such as music performance, and error processing in general.

In these studies, highly-trained pianists (mostly piano students or graduated pianists from music conservatories) were asked to perform either right-hand excerpts from pieces of the classical piano literature (Ruiz et al., 2009, 2011; Strübing et al., 2012), or bimanually scales and fingering patterns (Maidhof et al., 2009). To exclude the influence of visual feedback and to provoke pianists committing errors, these sequences were performed without visual feedback from the keys and hands, and at relatively fast tempos, ranging from 125–360 ms inter-onset intervals (IOIs). In addition to the EEG, the timing of note on- and offsets as well as an estimate of the key press velocity (corresponding to the loudness of the auditory feedback of a keystroke) were recorded in form of musical instrument data interface (MIDI) events.

On a behavioral level, two consistent findings across these studies were reported. First, erroneous keystrokes were performed with a lower velocity than correctly performed keystrokes, independently of whether correct keystrokes were performed with the same hand (at other positions in the score; Ruiz et al., 2009, 2011; Strübing et al., 2012), or simultaneously with the other hand (Maidhof et al., 2009). Furthermore, the analysis of bimanual sequence production revealed that the velocity of correct keystrokes when an error was present in the other hand did not differ from the velocity of correct keystrokes when no error was present. This indicated that the velocity of erroneous keystrokes in one hand did not influence the velocity of the simultaneous correct keystroke (Maidhof et al., 2009). Second, the IOIs, measured from the previous note to the current note, of incorrect keystrokes were increased compared to correct keystrokes, i.e., errors were produced slower than correct key presses (“pre-error slowing”). Although not analyzed by Maidhof et al. (2009), the studies by Ruiz et al. (2009, 2011) and Strübing et al. (2012) consistently reported also post-error slowing, i.e., the IOIs of correct notes directly following errors (measured from the current note to the next one) were prolonged. When stimuli were produced bimanually, the IOIs of both, the correct key press and the simultaneous incorrect key press were prolonged, compared to IOIs when no error was present in either hand. That is, whereas the keystroke velocity differed between incorrect and simultaneous correct keystrokes performed with two hands, timing affected both hands.

The study by Ruiz et al. (2009) employed also a condition in which pianists performed in the absence of auditory feedback. However, behavioral indices of “muted” piano performances did not differ in terms of timing, key press velocities, error rates, and behavioral features of errors; that is, results of silent piano performances showed pre-and post-error slowing, as well as reduced keystroke velocities during erroneous keystrokes. These findings are consistent with previous studies showing that the complete absence of auditory feedback seems to have no effects on performances of well-learned piano pieces (Finney, 1997; Finney and Palmer, 2003; Pfordresher, 2003; Pfordresher and Palmer, 2006; see also section below).

**Figure 1:**
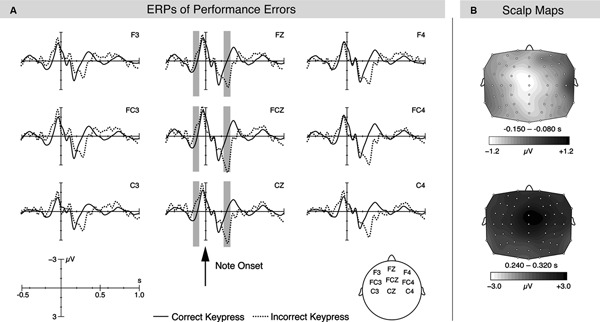
**(A)** Grand-averaged ERPs for correct and one-handed incorrect key presses during bimanual performance of technical piano exercises. The arrow indicates the note onset determined by the MIDI signal and hence the onset of auditory feedback. The gray areas show the time windows used for statistical analysis (−150 to −80 ms and 240–320 ms). **(B)** shows the scalp distributions for the difference potential for correct keystrokes subtracted from incorrect keystrokes. Figure taken from Maidhof et al., 2009.

On a neurophysiological level, these studies (Maidhof et al., 2009; Ruiz et al., 2009; Strübing et al., 2012) consistently reported that ERPs elicited during incorrect and correct key presses showed a negative difference occurring prior to the note onset (determined by the onset of the MIDI signal; see Figure 1). Thus, ERPs differed before the erroneous movement was fully executed and before auditory feedback was available (this effect was termed pre-error negativity or pre-error-related negativity (pre-ERN). The latency of this effect appears to be influenced by the tempo with which pianists produced the sequences: when participants played with a relatively slower tempo (IOI of around 360 ms), the difference occurred earlier around 150–80 ms prior to note onset (Maidhof et al., 2009), whereas the difference occurred somewhat later around 70–20 ms prior to note onset when stimuli were produced in a relatively fast tempo (IOI of around 125 ms; Ruiz et al., 2009, 2011; Strübing et al., 2012). One might speculate that during slower performance tempi, there is more time to prepare, initiate, and execute the following keystroke; consequently, error detection and/or error correction mechanisms can start at an earlier stage, resulting in the different latency of the pre-error negativity during slower performances. Alternatively, one might speculate that the latency differences of the early negativity occurring during slower and faster tempi are due to an artifact of the ERP analyses. Because one could assume that ERPs of the wrong notes overlap with the ERPs of the previous notes, differences in the performed tempo might result in latency differences of the sum amplitude of this overlap, possibly causing a pseudo-shift of pre-ERN latencies. However, in the study of Ruiz et al. (2009), a symbolic resonance analysis was performed, which can be used to disentangle possible overlapping brain responses due to short IOIs (Beim Graben and Kurths, 2003). The results of this analysis confirmed the ERP analysis in the time window of the pre-ERN. Thus, it seems unlikely that the different latencies of the pre-ERN obtained during varying performance tempi are only due to the above mentioned ERP artifact. Similarly, the pre-ERN might be influenced by the different tempi of correct and incorrect notes. To further exclude this possibility, future experiments could compare a subset of correct notes which are performed with a tempo that is comparable to that of incorrect notes. If the pre-ERN would still be elicited, it would indicate that the different tempi of correct and incorrect notes can have only a minor influence on the pre-ERN.

Despite the latency differences, the ERP effect showed consistently a (fronto-) central topographical distribution, and was estimated to be generated by brain structures in the rostral part of ACC (Ruiz et al., 2009), consistent with an explanation in terms of error-related processes (Ridderinkhof et al., 2004). Separate analysis of errors committed with the right or left hand did not show any lateralization effects (Maidhof et al., 2009), which indicates that the ERP difference is not due to low-level motor-related processes which could be the cause of the error, but rather due to cognitive processes of error detection and/or correction: simpler motor-related execution processes would be expected to occur lateralized or bilaterally when averaged across left- and right-hand errors.

Interestingly, the pre-error negativity was also elicited in the absence of auditory feedback, and its amplitude did statistically not differ between the two feedback conditions (although it occurred in the condition without feedback around 50–0 ms prior to note onset). However, the results of a symbolic resonance analysis and the ERP difference waves of Ruiz et al. (2009) suggest that the pre-error negativity elicited in the absence of auditory feedback might actually consist of two superimposed components of slightly different amplitudes. Thus, future work is needed to disentangle the exact differences between error processing with and without auditory feedback. Nevertheless, it is important to note here that these findings indicate that not the processing of auditory input (which differs between correct and incorrect key presses) can account for the pre-error negativity.

Another finding of the above mentioned studies was that incorrect keystrokes elicited, compared to correct keystrokes, an increased positivity around 250 ms after the onset of errors (see also Figure 1). This potential showed a fronto-central scalp topography and resembles the Error Positivity (Pe) or the P3a, which is frequently observed in the context of error processing during choice-reaction time tasks (Overbeek et al., 2005) and during the processing of novel or unexpected events (Polich and Kok, 1995; Comerchero and Polich, 1999; Polich, 2007). However, it has also been suggested that the Pe and P300 might actually reflect similar neuronal and functional processes (Overbeek et al., 2005; Ridderinkhof et al., 2009), paralleling the suggestion that the ERN and the novelty-related N2 ERP component share an underlying neural network (Wessel et al., 2012). The latency of the Pe seemed to be less influenced by the performance tempo, but by the presence/absence of auditory feedback: in the condition without auditory feedback, it already occurred around 200 ms and its amplitude was decreased, compared to when auditory feedback was available (Ruiz et al., 2009). This component is probably related to the conscious recognition of an error (“error-awareness hypothesis”, e.g., Nieuwenhuis et al., 2001). In addition, the conscious recognition of an error might also lead to the adaptation of response strategies (“behavior-adaptation hypothesis”, Hajcak et al., 2003), which could be reflected in the post-error slowing after performance errors (Ruiz et al., 2009, 2011; Strübing et al., 2012). Finally, the recognition of errors might also lead to affective responses, including autonomic changes, e.g., in heart and respiration rate, and sweat production (see also the “affective processing hypothesis”, summarized in Overbeek et al., 2005). These changes might be especially pronounced in musicians, considering that their expertise is the product of thousands of hours of deliberate practice (Ericsson et al., 1993; Ericsson and Lehmann, 1996; Sloboda et al., 1996), and they could be particularly obstructive in a concert situation, because increased amounts of sweat might constrain the proper manipulation of the instrument. In addition, these physiological changes might conflict with the emotional aspects of a performance. However, empirical studies investigating the affective responses following performance errors are lacking, and it might be interesting to investigate the various factors, including auditory feedback, modulating possible affective responses.

The ERP effect occurring prior to the execution of performance errors (and before auditory feedback was available) suggests that errors were detected before they were committed. Considering that it is unlikely that fast sequential behaviors such as piano performance are planned and executed element by element (i.e., note by note; see e.g., Lashley, 1951), it is reasonable to assume that also error detection occurs in a predictive manner.

Borrowing from recent models of motor control (Wolpert et al., 1995; Miall and Wolpert, 1996; Wolpert et al., 1998; Desmurget and Grafton, 2000; Wolpert and Ghahramani, 2000), it has been argued that error detection during piano performance is based on internal forward models. These models consist basically of a motor controller, which sends motor commands to an effector, resulting in overt movements. In addition, sensory systems receive for example proprioceptive, tactile, and visual feedback about the ongoing movements. The forward model receives an efference copy of motor commands. Based on learned associations between motor commands and their sensory consequences and the current state of the body, it generates predictions about the next state of the effector as well as about the sensory consequences of a movement. A comparator element identifies if there is a difference between the actual and predicted sensory consequences, and triggers an error signal in case of a mismatch. This error signal can in turn be used for corrective modulation of motor commands and to rapidly correct ongoing actions (for example, in a pointing task, it was reported that participants made corrections to their hand trajectory already 30 ms after start of the movement; van Sonderen et al., 1989). The pre-error slowing and the decreased key press velocity during piano performance errors (Maidhof et al., 2009; Ruiz et al., 2009, 2011; Strübing et al., 2012) might have been the result of this corrective modulation of the motor command.

Although internal forward models provide a theoretical account for the ERP effect prior to performance errors and the behavioral findings, it remained unclear whether the pre-error negativity reflects an error signal itself or is associated to the implementation of behavioral adjustments. This question was investigated by a study that looked at interactions between different brain regions during the commission of errors (Ruiz et al., 2011). More specifically, that study investigated the interaction, in terms of neural oscillatory synchronization, between the posterior frontomedial cortex (pFMC) (particularly the ACC, where the pre-error negativity is presumably generated) and the lateral prefrontal cortex (lPFC). The rationale behind this approach was based on recent theories of action monitoring postulating that these regions interact as part of a dynamic loop during goal-directed behavior, with the pFMC involved in action monitoring, and the lPFC involved in cognitive control (MacDonald et al., 2000; Botvinick et al., 2001; Ridderinkhof et al., 2004). In that view, the ACC detects unfavorable outcomes, conflicts and errors, and signals the need for increased cognitive control to the lPFC, which then implements performance adjustments (Miller, 2000; Cavanagh et al., 2009; Wittfoth et al., 2009). The results of the study by Ruiz et al. (2011) showed increased theta and beta band oscillations prior to errors (and also extending until after errors) over the pFMC. Importantly, the phase synchronization in the beta band (ca. 14–18 Hz) between electrodes F4 and FCZ (corresponding to lPFC and pFMC, respectively) increased around 100 ms prior to errors, and this increase was associated with more efficient corrective mechanisms. The latter was indicated by a positive correlation between the synchronization index and the decrease in loudness of errors, and by a negative correlation between the synchronization index and pre-error slowing. Based on these findings, it was suggested that the error signal is indexed by pre-error beta and theta oscillations of pMFC, and this information is conveyed to the lPFC, which then implements behavioral adjustments (like slowing down the next key press).

## Auditory feedback and motor performance

The above summarized findings strongly indicate that auditory feedback is not a prerequisite for the detection of errors committed during music (piano) performances. An interesting question is then what role auditory feedback actually plays during music production. A common approach to study this question is to look at motor performance in response to manipulations of the auditory feedback (in the so-called “altered auditory feedback” paradigm). Motor performance is usually quantified in terms of error rates, IOIs, and timing variability, all of which are conceptualized to represent deviations from the intended performance.

In line with the notion that auditory feedback plays a minor role for error monitoring, several studies showed that the absence of sound had negligible effects on performances of trained pianists (Finney, 1997; Repp, 1999; Finney and Palmer, 2003), as well as on untrained pianists (Pfordresher, 2005). In contrast to these negligible effects on piano performance, it has been shown that error detection and correction crucially depend on the presence of auditory feedback, for example, during cello performances (Chen et al., 2008). On the other hand, specific alterations of feedback can profoundly disrupt piano performance (Finney, 1997; Pfordresher and Palmer, 2002; Pfordresher, 2003; Furuya and Soechting, 2010). Furthermore, there appears to be a dissociation between two components of auditory feedback, namely pitch and timing: pitch manipulations of auditory feedback resulted mainly in higher error rates, but did not influence timing (especially in serial shifts, i.e., when the feedback matches an event intended at a different location in a sequence; Pfordresher, 2003; Pfordresher and Palmer, 2006); in contrast, delays between key press and onset of auditory feedback increased timing variability and IOIs, but error rates were only marginally increased. More specifically, the disruption from feedback delays seems to depend on the relative phase (rhythmic) relationship between keystroke and feedback onset (e.g., Pfordresher and Palmer, 2002; Pfordresher, 2003; Pfordresher and Benitez, 2007; for an account how these sensorimotor associations might be learned, see Pfordresher, 2012).

Recently, there were also some attempts to study the neural mechanisms underlying the processing of auditory feedback manipulations during piano performance. In these studies (Katahira et al., 2008; Maidhof et al., 2010), pianists and participants with no formal music training performed sequences and were provided occasionally (< 5%) with manipulated auditory feedback, so that the pitch of one tone was lowered by one semitone (Maidhof et al., 2010) or shifted up by one semitone (Katahira et al., 2008).

Results showed that ERPs of correct key presses with feedback manipulations, compared to correct key presses with the corresponding correct feedback, elicited a negativity that was maximal around 200 ms after tone onset and showed a fronto-central scalp distribution, and that was present only in musicians. Although termed differently by Katahira et al. (2008), it was argued that this potential reflects mainly a feedback error-related negativity (a potential usually observed after negative performance feedback indicating loss or punishment in time estimation tasks, guessing tasks, and gambling tasks; Miltner et al., 1997; Hajcak et al., 2005, 2007), with potential contribution of MMN/ERAN potentials (Maidhof et al., 2010). The negativity was followed by two positive potentials, resembling the P3a and P3b (or Pe).

Because the perception of manipulated feedback presumably elicits not only action-related processes, but also cognitive processes related to the perception of acoustic deviants, the ERPs during the production of musical sequences were also compared to ERPs elicited during the mere perception of the same stimulus material. Results of this comparison showed that the negative potential is also elicited during perception, but clearly reduced (Katahira et al., 2008; Maidhof et al., 2010). Therefore, it was assumed that similar (expectancy) mechanisms operate during production and perception of music, but that the intention and action of producing a certain auditory effect by performing key presses influences the processing of the unexpected auditory input.

## Outlook

This brief review summarized recent investigations into the neural mechanisms underlying error and feedback processing during music performance. The few mentioned studies provided some initial insights; however, because this is a relatively new line of research, many questions remain naturally unsolved. Some of these questions–in the hope that they will motivate further research in this domain–will be addressed in the following.

With regards to error monitoring, information about the kinetic and kinematic features of movements were lacking, and neural correlates could only be related to performance parameters yielded at discrete time points like tone on- and offsets (indicated by the MIDI signal, providing information about when a key was pressed down or released) or key velocities. However, information about different movements or movement stages (e.g., movement onset, touching the surface of a piano key, downward movement during the actual key press) could help to disentangle the different contributions of tactile and proprioceptive feedback for error monitoring processes. Detailed movement data with high spatial and temporal accuracy could be provided by so-called motion capture techniques. Studies using these techniques investigated a variety of research questions, like the role of tactile feedback in timing accuracy during piano and clarinet performance (Goebl and Palmer, 2008; Palmer et al., 2009), disruptive effects of delayed auditory feedback during rhythm production and the role of the ongoing movement trajectory (Pfordresher and Bella, 2011), the effect of tempo on finger kinematics in pianists (Bella and Palmer, 2011), or movements involved in emotional expressions (Livingstone et al., 2009) and as cues for other performers in ensemble performance (e.g., Keller and Appel, 2010). Thus, the simultaneous recording of electrophysiological data and movement data could lead to a more behaviorally informed brain research that could help to answer important questions in the domain of error monitoring (for a setup combining EEG, MIDI, and motion capture for investigating music performance, see Maidhof et al., in press; for a general-purpose setup, see Makeig et al., 2009). Thus, new methodological developments will also advance our understanding of neural processes of error monitoring.

With regards to the nature of performance errors, it has been shown that the frequency and kind of errors are influenced by the musical structure (e.g., musical phrase structure) and underlying planning processes, and that not all errors are equally important for listeners (Palmer and van de Sande, 1993, 1995; Repp, 1996). However, we do not know if the neural correlates of different types of errors occurring in different musical contexts differ from each other. In addition, the neural mechanisms underlying other types of errors such as fingering errors remain elusive. It would thus be interesting to investigate the influence of the musical context on the neural correlates of error processing, and thus to know if the same neural mechanisms underlie different error types occurring in different musical contexts. For example, are errors at phrase boundaries processed differently than errors within phrases, or are there any differences between the neural correlates of errors with different metrical accentuations? How are omission errors (i.e., if a note in the score was not played) processed, given that auditory ERPs can be elicited even when a stimulus is omitted from a regular auditory pattern (so-called omission-evoked potentials), and given that these potentials can be influenced by expectancy and musical training (Jongsma et al., 2005)? Because fingering in piano performance seems to depend, at least partially, also on the metrical and melodic structure (Clarke et al., 1997; Parncutt et al., 1997), it might also be interesting to compare the neural correlates of fingering errors at different positions in a performance. The main question is therefore to reveal any interactions between the error processing system and higher-level processing of musical regularities.

Similarly, with regards to auditory feedback, we do not know if and what influence the musical context and its structure has on the processing of auditory feedback (for example, is auditory feedback of metrically weak notes equally important as metrically strong notes), and it would be interesting to reveal any interactions between the processing of auditory feedback and higher-level musical regularities. This could be achieved by comparing the neural responses to careful manipulations of feedback (of varying sizes in pitch or temporal delay) at different positions within a sequence.

Another topic for further research could be investigations into the affective responses to errors. Although there has been progress studying affective responses during the performing of choice reaction-time tasks (e.g., Fiehler et al., 2003; Hajcak et al., 2003; Critchley et al., 2005), this issue has not yet been investigated for music performance. Furthermore, individual differences in error processing and in affective responses to errors remain elusive.

The ultimate goal would be to arrive at a better understanding of error monitoring and action control during music performance. This knowledge could help to learn how errors during performance could be prevented in the first place, but probably even more important, also to improve skill learning. To reach this goal, a fruitful approach could be to look for similarities to errors in the speech domain. Recently, Hickok (2012) proposed a “hierarchical state feedback control model” of speech production, which is partly based on findings from speech error analysis. These findings indicated a hierarchical organization of speech production, which is similar to findings in music performance (Palmer and van de Sande, 1993, 1995). The speech production model of Hickok (2012) incorporates these hierarchies by postulating multiple levels of control that interact during speech production. Error detection and correction are realized by postulating forward predictions that are compared to auditory and somatosensory feedback. Thus, it is conceivable that a future model of error processing during music performance can benefit from theoretical work in the speech domain.

Finally, music performance is mostly a social situation, with different performers and listeners participating in an interactive situation (for a recent ERP study investigating feedback processing in a piano duet situation, see Loehr et al., 2013; for a study investigating alpha oscillations and empathy in saxophone quartets, see Babiloni et al., 2012; for a review about ensemble music, see Palmer, 2012). Hence, the question for future research will also be if and how models of individual behavior like error monitoring and feedback processing can be applied to such contexts.

## Conflict of interest statement

The authors declare that the research was conducted in the absence of any commercial or financial relationships that could be construed as a potential conflict of interest.
